# Management of Pregnant Women with Mental Disorders Requires Attention to Gestational Diabetes Mellitus

**DOI:** 10.1089/whr.2023.0112

**Published:** 2024-02-23

**Authors:** Kumiko Fujii, Shunichiro Tsuji, Mayuko Ono, Haruka Yamazaki, Takashi Murakami, Yuji Ozeki

**Affiliations:** ^1^ Department of Psychiatry, Shiga University of Medical Science, Otsu, Japan.; ^2^ Department of Obstetrics and Gynecology, Shiga University of Medical Science, Otsu, Japan.

**Keywords:** pregnant women with psychiatric complications, antipsychotics, antidepressants, gestational diabetes mellitus

## Abstract

**Background:**

Psychiatric interventions may be required during pregnancy. In the aspect of the management of psychiatric symptoms and the consideration of the need for pharmacotherapy, possibly to manage the effects on the fetus, pregnant women with mental disorders are considered high risk as other physical illnesses.

**Objective:**

We investigated the characteristics of pregnant women with psychiatric disorders compared with high-risk pregnant women with physical illnesses at our university hospital and the effects of psychotropic drug use on pregnant women with mental disorders and their children.

**Materials and Methods:**

In a multivariate analysis of 1282 pregnant women, excluding those with multiple pregnancies who gave birth at our hospital between January 2017 and the end of December 2019, we evaluated the effects of mental disorders and the use of psychotropic drugs throughout at least the third trimester up to the day of delivery on obstetric complications and infants. All data were collected retrospectively.

**Results:**

Ninety-nine pregnant women had mental disorders and 62 took psychotropic drugs. Among multiple factors, pregnant women with mental disorders were associated with significantly higher rates of smoking and gestational diabetes mellitus (GDM) and significantly lower child abnormalities. The cause or effect was difficult to determine; however, the use of antipsychotics or antidepressants was also significantly associated with GDM, while psychotropic use was not related to any of the other factors investigated in this study.

**Conclusions:**

Attention to GDM might be important in the management of pregnant women with mental disorders.

## Introduction

### Pros and cons of psychotropic medications for pregnant women

In schizophrenia and bipolar disorder, discontinuation of the medication frequently worsens symptoms. In prepregnancy depression, the risk of relapse due to discontinuation of medication is high, with relapse rates reported to be 68% and 26% in pregnant women who discontinued and continued the medication, respectively, even when the medication was in remission^1^. Relapse of mental illness may be associated with suicide in worst-case scenarios. Psychiatric disorders such as depression have been associated with an increased risk of suicide in pregnant women^2^.

The effects of the use of antipsychotic and antidepressant drugs during pregnancy in newborns have been reported^3,4^. However, these effects may be individualized, and some pregnant women may have concerns about congenital malformations^5^. One of the disadvantages is the risk of withdrawal syndrome in newborns^6^.

Despite these facts, there are maternal advantages in continuing to take medication in patients with mental disorders, including during the postpartum period. In addition to pharmacotherapy, the mental state of the mother may have various effects on the fetus and newborn baby ^7^; maternal depression has been associated with mother–child bonding^8^.

### Pregnant women with perinatal psychiatric complications

Despite this disadvantage, such as some pregnant women may have concerns about congenital malformations^5^ and one of the risks is withdrawal syndrome in newborns^6^, the medication can help stabilize the mental state of a woman during pregnancy, protect maternal safety, and reduce the deterioration of mother–infant bonding.

Pharmacotherapy is important to prevent relapses in patients with mental disorders. In particular, for patients with schizophrenia and bipolar disorder, continued medication is important to prevent relapse. For bipolar disorder, recurrence has been reported to be significantly higher when medication is discontinued during pregnancy^9^. Furthermore, for depression, the recurrence rate was reported to be 26% when the medication was continued during pregnancy, but 68% when it was discontinued, with a higher recurrence rate in the discontinuation group^1^. Therefore, the overall benefits of psychotropic medications may outweigh their disadvantages in postpartum childcare.

In Japan, pregnancies complicated by mental illness are classified as high risk due to the need for appropriate intervention in the mental health of pregnant women, as well as the need to prevent the deterioration of mental disorders during the perinatal and neonatal periods. Pregnant women with mental disorders are often admitted to comprehensive perinatal medical centers with their psychiatric departments. However, many high-risk cases, in addition to mental disorders, such as maternal and fetal complications, are also referred to general perinatal medical centers.

### About our general perinatal center

Our area is a province (region) with a population of 1.41 million, and there are four perinatal medical centers for pregnant women with complications. Our university hospital is a comprehensive perinatal medical center with a psychiatric department. In our hospital, pregnant women are referred not only for psychiatric complications, but also for physical complications in the mother, such as gestational hypertension, placenta previa, threatened premature delivery, severe hyperemesis gravidarum, eclampsia, history of preterm delivery, *etc.*, and apparent fetal abnormalities. From this perspective, the facility is also for pregnant women with maternal and fetal abnormalities as well as mental disorders.

Therefore, we conducted a survey to determine what should be considered when managing pregnancies complicated by mental disorders compared with other high-risk pregnancies in a general perinatal center where various high-risk pregnant women and cases of fetal abnormalities are concentrated.

### Drug selection during pregnancy and the current situation

Anxiety is associated with drug therapy during pregnancy due to its effects on the fetus. In particular, pregnant women often have vague fears that drug therapy may cause fetal malformations.

However, several drugs require special attention during pregnancy due to teratogenic issues. In Japan, lithium carbonate is contraindicated during pregnancy, and valproic acid should be avoided if possible. The risk of cardiac malformations has been reported for lithium carbonate^10^ and for valproic acid, fatal malformations^11^ and effects on intellectual function of children exposed *in utero*^12^ have been reported. In Japan, most of psychotropic drugs, such as antipsychotics, antidepressants, and GABA receptor agonists, are available for use during pregnancy if the need outweighs the risks. However, due to the individual differences between mothers, fetuses, and newborns, detailed observation and careful judgment are required.

### Background of the present analysis

This study was carried out in our university hospital with a previously described background. This survey was conducted to clarify what should be considered when treating pregnant women with mental disorders compared with other high-risk pregnancies in comprehensive perinatal medical centers of high-risk pregnancy, where various high-risk pregnant women and cases of fetal abnormalities are concentrated.

## Materials and Methods

This study was carried out as an opt-out basis after receiving approval from the Ethics Review Office of Shiga University of Medical Science. Data were collected from medical records as a retrospective observational study. The analysis was performed by a person different from the one who collected the data. We collected data from the patient's medical records. Regarding gestational diabetes mellitus (GDM), based on urinalysis and blood glucose test by the obstetrician–gynecologist, an oral glucose tolerance test was performed in suspicious cases, and if the values met the criteria for GDM, the patient was diagnosed with GDM and managed by a diabetes endocrinologist at our hospital. Patients with a history of GDM in previous pregnancies are checked, but if their values do not meet the criteria for GDM in this pregnancy, they are not diagnosed with GDM. Patients with prepregnancy diabetes mellitus were excluded.

In 1282 pregnant women who gave birth in our hospital between January 2017 and the end of December 2019 at Shiga University of Medical Science Hospital, excluding multiple pregnancies, the presence or absence of mental disorders (including epilepsy), and whether the use of psychotropic drugs throughout at least the third trimester up to the day of delivery had an impact on obstetric complications (hypertension and GDM), the number of weeks of gestation, fetal growth failure, infant birthweight, admission to the neonatal intensive care unit (NICU), fetal abnormalities such as cardiovascular, urological, gastrointestinal, and oral abnormalities, fetal dysfunction, Apgar score, amount of blood loss at delivery, umbilical artery blood pH, and mode of delivery were assessed in a multivariate analysis.

Epilepsy is a neurological disorder that is usually treated with antiepileptic drugs. Patients with epilepsy are occasionally treated in the Department of Psychiatry in Japan. As antiepileptics are also used in patients with bipolar disorder, we included patients with epilepsy in this study.

Only those who habitually smoked were considered smokers, and those who continued smoking throughout pregnancy were analyzed.

In Japan, cesarean sections are generally indicated for certain complications. The average rate of cesarean section in Japan is 20%, while that of our hospital is 40%. The number of pregnant women for whom a cesarean section is indicated is higher than the average in our hospital. Cases with maternal abnormalities such as gestational hypertension, placenta previa, threatened premature delivery, severe hyperemesis gravidarum, eclampsia, history of preterm delivery, fetal abnormalities suspected by fetal diagnosis, and stunted growth are referred to our Department of Obstetrics and Gynecology.

All statistical analyses were performed with SPSS version 25 (IMB Corp, Armonk, NY).

## Results

Ninety-nine pregnant women had mental disorders, 62 took psychotropic drugs throughout at least the third trimester up to the day of delivery, and 70 were taking medication when they realized their pregnancy, which meant that they had taken it during the first trimester. Table 1 lists the demographic characteristics of the participants. Table 2 lists the diagnoses of the mental diseases, including their coexistence. Except for epilepsy, all diagnoses were made according to the Diagnostic and Statistical Manual of Mental Disorders, fifth edition (DSM-5) criteria. In addition, comorbidities and number of patients are listed in the Supplementary Table S1.

**Table 1.** Demographic Characteristics of Pregnant Women

	With mental disorder (***n*** = 99)	No mental disorder (***n*** = 1183)	***p***
Maternal age	32.29 ± 5.9	33.44 ± 5.31	0.220
Number of births	1.71 ± 1.94	1.66 ± 1.84	0.538
Gestational age	38.2 ± 3.0	37.5 ± 2.7	0.005
Cesarean section (with/without)	30/69	529/654	0.006
Birthweight of baby	2900 ± 453	2764 ± 623	0.005
Sex of the baby (M:%)	50 (50.1)	600 (50.7)	1.000
Apgar score at 1 minutes	7.8 ± 1.1	7.7 ± 1.4	0.125
Apgar score at 5 minutes	8.8 ± 0.6	8.8 ± 0.8	0.865
Volume of blood loss	787.6 ± 477	875.8 ± 551	0.144
Umbilical cord artery blood pH	7.29 ± 0.06	7.28 ± 0.07	0.380
Admission to NICU (w[%]/wo[%])	15 (15.2)/84 (84.8)	347 (29.3)/836 (70.7)	<0.001
Smoking (w[%]/wo[%])	10 (10.1)/89 (89.9)	20 (1.7)/1163 (98.3)	<0.001
Hypertension of mother (w[%]/wo[%])	5 (5.1)/94 (94.9)	106 (9.0)/1077 (91.0)	0.262
Gestational diabetes mellitus (w[%]/wo[%])	19 (19.2)/80 (80.8)	120 (10.1)/1163 (89.9)	0.010
Fetal growth abnormality (w[%]/wo[%])	7 (7.1)/92 (92.9)	114 (9.6)/1069 (90.4)	0.478
Fetal abnormality (including fetal dysfunction) (w[%]/wo[%])	4 (4.0)/95 (96.0)	172 (14.5)/1011 (85.5)	0.002

NICU, neonatal intensive care unit; w/wo, with/without.

**Table 2.** Diagnosis of All Patients with Mental Disorders (*n* = 99) and Those with Psychotropic Medications Throughout at Least the Third Trimester Up to the Day of Delivery (*n* = 62) (Including Coexistence)

	Total	Patients with psychotropic medications	Age (total)
Schizophrenia	9	8	32.4 ± 5.3
Bipolar and related disorder	9	7	32.5 ± 6.3
Depressive disorder	26	18	32.5 ± 5.0
Panic disorder	18	13	33.8 ± 5.3
Anxiety disorder	7	4	31.0 ± 5.3
Feeding and eating disorder	4	3	31.5 ± 5.9
Obsessive compulsive disorder	3	1	32.5 ± 5.0
Epilepsy	17	12	32.8 ± 6.0
Others, including neurodevelopment disorder	16	5	31.8 ± 7.9
Total	99	62	

Except for epilepsy, diagnoses are made according to the DSM-5.

DSM-5, Diagnostic and Statistical Manual of Mental Disorders, fifth edition.

Table 3 lists the types of psychotropic drugs that were administered in this study. In addition, 31 patients taking multiple psychotropic medications were also included. Data are shown in Supplementary Table S2. Their mental conditions were sufficiently stable to continue their pregnancies. As the number of cases was too small to statistically examine the efficacy of each drug, we divided this study into two parts according to whether the patients were taking the drugs. Nineteen pregnant women were diagnosed with mental disorders and GDM; their diagnoses and medications are shown in Supplementary TableS3.

**Table 3.** Types and the Total Number of Medications Taken by Pregnant Women with Mental Disorders (*n* = 62)

Type of medications	Total number
Antipsychotics	26
Antidepressants	25
GABAergic receptor agonist	33
Antiepileptics	17
Orexin receptor antagonist	5

Includes patients taking multiple types of psychotropic medications.

Table 4 lists the impact of psychiatric complications according to logistic regression analysis. Pregnant women with mental disorders had significantly higher rates of smoking and there is a significant relationship between pregnant women with mental disorders and GDM, while the number of abnormalities in their children was significantly lower.

**Table 4.** The Impact of Psychiatric Complications: the Result of Logistic Regression Analysis

	PRC (95% CI)	Wald	Odds ratio (95% CI)	***p***
Maternal age	−0.04 (−0.08 to −0.002)	4.09	0.96 (0.923 to 1.00)	0.04
Number of births	0.09 (−0.17 to 0.34)	0.46	1.09 (0.847 to 1.41)	0.50
Gestational age	0.08 (−0.06 to 0.21)	1.30	1.08 (0.945 to 1.24)	0.25
Cesarean section	−0.36 (−0.85 to 0.14)	2.01	0.70 (0.427 to 1.15)	0.16
Birthweight of baby	0.00 (0.00 to 0.00)	0.47	1.00 (1.00 to 1.00)	0.83
Sex of the baby	0.07 (−0.36 to 0.50)	0.09	1.07 (0.694 to 1.65)	0.76
Apgar score at 1 minutes	−0.05 (−0.33 to 0.22)	0.14	0.95 (0.719 to 1.25)	0.71
Apgar score at 5 minutes	−0.18 (−0.55 to 0.20)	0.86	0.84 (0.575 to 1.22)	0.36
Volume of blood loss	0.00 (0.00 to 0.00)	0.86	1.00 (1.00 to 1.00)	0.35
Umbilical cord artery blood pH	0.08 (−3.35 to 3.52)	0.00	1.09 (0.035 to 33.82)	0.96
Admission to NICU	−0.57 (−1.3 to 0.16)	2.37	0.56 (0.272 to 1.17)	0.12
Smoking	2.07 (1.19 to 2.94)	21.36	7.88 (3.285 to 18.92)	<0.001
Hypertension of mother	−0.33 (−1.27 to 0.62)	0.46	0.72 (0.28 to 1.86)	0.50
Gestational diabetes mellitus	0.75 (0.19 to 1.32)	6.86	2.12 (1.209 to 3.73)	0.01
Fetal growth abnormality	−0.32 (−1.15 to 0.51)	0.57	0.73 (0.316 to 1.67)	0.45
Fetal abnormality	−1.01 (−2.07 to 0.05)	6.38	0.26 (0.089 to 0.74)	0.01

Pregnant women with psychiatric complications had significantly higher rates of smoking and GDM and significantly lower rates of NICU admission for their babies.

CI, confidence interval; GDM, gestational diabetes mellitus; PRC, partial regression coefficient; *p* value, significant probability.

Logistic regression analysis with GDM as the dependent variable showed that taking antipsychotic or antidepressant medications throughout at least the third trimester up to the day of delivery had a significant relationship with GDM (Table 5); however, psychotropic medication use was not correlated with any of the other factors investigated in this study. Maternal age was also significantly associated with GDM.

**Table 5.** The Impact of Gestational Diabetes Mellitus: the Result of Logistic Regression Analysis

	PRC (95% CI)	Wald	odds ratio (95% CI)	***p***
Maternal age	0.07 (0.04 to 0.11)	15.31	1.08 (1.04 to 1.12)	<0.001
Number of births	0.03 (−0.18 to 0.24)	0.08	1.03 (0.84 to 1.27)	0.78
Gestational age	−0.01 (−0.12 to 0.10)	0.03	0.99 (0.89 to 1.11)	0.87
Cesarean section	−0.08 (−0.48 to 0.32)	0.14	0.93 (0.62 to 1.38)	0.71
Birthweight of baby	0.00 (0.00 to 0.00)	0.10	1.00 (1.00 to 1.00)	0.75
Sex of the baby	0.04 (−0.33 to 0.41)	0.05	1.04 (0.72 to 1.51)	0.83
Apgar score at 1 minutes	−0.07 (−0.28 to 0.14)	0.42	0.93 (0.75 to 1.15)	0.52
Apgar score at 5 minutes	0.03 (−0.40 to 0.46)	2.01	1.37 (0.89 to 2.11)	0.16
Volume of blood loss	0.00 (0.00 to 0.00)	0.25	1.00 (1.00 to 1.00)	0.62
Umbilical cord artery blood pH	−0.36 (−3.28 to 2.57)	0.06	0.70 (0.04 to 13.06)	0.81
Admission to NICU	−0.49 (−1.09 to 0.10)	2.62	0.61 (0.34 to 1.11)	0.11
Smoking	0.42 (−0.75 to 1.60)	0.50	1.53 (0.47 to 4.94)	0.48
Hypertension of mother	−0.42 (−1.19 to 0.36)	1.10	0.66 (0.30 to 1.43)	0.29
Mental disease of mother	−0.15 (−1.12 to 0.83)	0.09	0.86 (0.33 to 2.28)	0.77
Taking antipsychotics	1.39 (0.12 to 2.66)	4.62	4.02 (1.13 to 14.26)	0.03
Taking antidepressants	2.11 (0.70 to 3.51)	8.59	8.21 (2.01 to 33.55)	0.00
Taking GABAergic receptor agonist	−1.47 (−3.01 to 0.07)	3.52	0.23 (0.05 to 1.07)	0.06
Taking antiepileptics	1.14 (−0.33 to 2.62)	2.31	3.04 (0.72 to 13.72)	0.13
Taking orexin receptor antagonist	0.64 (−2.17 to 3.44)	0.20	1.89 (0.11 to 31.25)	0.66
Fetal growth abnormality	0.06 (−0.59 to 0.71)	0.03	1.06 (0.55 to 2.04)	0.86
Fetal abnormality	−0.09 (−0.71 to 0.53)	0.09	0.91 (0.49 to 1.69)	0.77

Antipsychotic and antidepressant medications are significantly associated with gestational diabetes mellitus.

Furthermore, analysis of covariance (ANCOVA) showed a significant difference between the four factors with GDM and mental disorder as covariates (Supplementary Table S4). The Tukey's HSD *post hoc* test (Tukey's honestly significant difference post hoc test) revealed the following results; admission to the NICU was significantly higher in pregnant women without GDM and mental disorders than in those with GDM and without mental disorders and those without GDM and with mental disorders. The prevalence of fetal abnormalities was significantly higher in pregnant women without GDM and mental disorders than in those without GDM and with mental disorders. Maternal age was significantly higher in pregnant women with GDM and without mental disorders than in those without GDM and mental disorders and in those without GDM and with mental disorders. Smoking was significantly higher in pregnant women with mental disorders and without GDM than in those without GDM and mental disorders and in those with GDM and without mental disorders.

Patients with both psychiatric disorders and GDM were not significantly different for any factor compared with the other groups. Because the number of them is small, there might not be characteristic findings. The results are presented in Supplementary Table S5.

## Discussion

In the present study, the number of abnormalities was lower in pregnant women with mental disorders. This could be due to selection bias, as many high-risk pregnant women with children who had abnormalities were included in the comprehensive perinatal center control group. Mental disorders and psychotropic drugs did not cause increased abnormalities in children. However, the prevalence of GDM is significantly higher in patients with mental disorders. The result is shown in Table 4. Taking antipsychotic or antidepressant medications throughout at least the third trimester up to the day of delivery had a significant relationship with GDM (Table 5). However, it should be added that this did not indicate that psychotropic medication is a risk for GDM, but only that there was an association between taking medication and the event of GDM.

Diabetes mellitus may also be an adverse effect of antipsychotics. A study reported that patients who continued to use antipsychotics were more likely to develop GDM than those who discontinued antipsychotics before pregnancy. Therefore, clinicians must be cautious about the diagnosis of GDM^13^. The choice of drug should be considered as it may vary depending on the type of drug. However, due to the small number of patients in each group, we were unable to analyze each drug type. The relationship between antidepressants and diabetes has been reported as follows:

Antidepressants have been associated with an increased risk of type II diabetes in nonpregnant women (21%–31% increase)^14^. These mechanisms include serotonin dysregulation, which regulates food intake and weight^15^, pancreatic beta-cell dysfunction^16^, and histamine 1 receptor antagonism^14^. The use of antidepressants with a strong affinity for histamine 1 receptors may increase the risk of GDM, although the report indicated that the inverse probability weighting did not indicate an increased risk^17^.

A systematic review and meta-analysis of antidepressant use during pregnancy and GDM reported that mothers exposed to antidepressants during pregnancy had a significantly increased risk of GDM, but also noted possible overestimation due to confounding factors^18^.

GDM is associated with postpartum depressive symptoms^19^ and increases the risk of subsequent depression and anxiety^20^. Prenatal depressive symptoms are also associated with the development of GDM^21^. A meta-analysis of the association between GDM and postpartum depression indicated that GDM may be a risk factor for postpartum depression^22^.

Because GDM increases perinatal risks, such as shoulder dystocia due to a large baby and neonatal hypoglycemia, the perinatal management of pregnancies complicated by mental disorders should consider abnormal glucose tolerance as well as other pregnant women.

Based on our findings, it may be possible to say not only that there is a possible association between psychotropic medication use and GDM, but also that pregnant women with GDM would be at risk for depression. In this research, due to the difficulty in determining the exact time of psychotropic medication and the onset of gestational diabetes, the association between the use of antipsychotics and antidepressants and gestational diabetes could not be concluded as a result or cause.

There are some limitations to this study. First, the severity of psychiatric symptoms in pregnant women was not assessed. However, their mental stability was maintained due to the evaluation and treatment of their psychiatrists, which resulted in their birth. Second, the possibility that the effects of mental disorders and psychotropic drugs were underestimated cannot be overlooked because mothers with fetal anomalies and nonpsychiatric complications were used for comparison. However, the presence of concurrent mental disorders and psychotropic medications did not result in significantly higher rates of fetal abnormalities or adverse effects in newborns.

Third, we were unable to investigate each antipsychotic drug individually and the risks may have differed for each drug. Fourth, the number of participants was small compared with several large surveys. However, we believe that it is important to clarify that in a single institution where various types of high-risk pregnant women are concentrated, it is important to pay attention when treating pregnancies complicated by mental illness versus other high-risk pregnancies.

Fifth, we were unable to assess the details of high-risk factors among pregnant women without psychiatric disorders as controls. Sixth, it was difficult to determine the exact time of use of psychotropic drugs and the onset of GDM; therefore, it was not possible to conclude that GDM occurred as a result of antipsychotic and antidepressant medications. Due to this association, pregnant women using antidepressants or antipsychotics should be carefully monitored for GDM.

Seventh, we were unable to assess body weight in this population. The association with body weight is an important point, as prepregnancy weight has been reported to be higher in the group using antipsychotic drugs^4^. There are still points to be evaluated, such as prepregnancy weight and weight gain throughout pregnancy, which is also a limitation of this study. Similarly, an association has been observed between obesity and GDM^23^. However, we were unable to analyze the association between obesity and GDM. This is another limitation of this study. One more limitation of this study is that the number of new users among the 62 patients who were taking psychotropic medications in the third trimester was not identified.

According to one study, taking psychotropic medications during pregnancy increases the risk of autism spectrum disorders ^24^. In the present study, the children were too young for assessment. However, reports^25^ and similar meta-analyses^26^ suggest that the mental disorder of the mother has a greater impact on the child than medication. A previous report also warned that it is difficult to determine whether this is due solely to the drug and that it is not recommended to increase anxiety beyond what is necessary^27^.

## Conclusions

Our results did not indicate a significant increase in the risk to newborns from women with mental disorders. However, due to the increased prevalence of GDM, it should be carefully managed.

Considering the symptoms and severity of an individual's condition, the need for medication should be considered when managing mental disorders during pregnancy. Although the number of participants was small compared with several large-scale surveys, we believe that it is important to clarify what merits attention when treating pregnant women with mental disorders compared with other high-risk pregnant women. We hope that this will be one of the studies used to address the “vague” concerns of pregnant women with mental disorders about the continued use of medication. Since we indicated just potential relationships between the events analyzed in this study, it would be of interest to calculate and investigate the incidence and risk of developing GDM in a similar cohort of patients in future studies.

## Supplementary Material

Supplemental data

Supplemental data

Supplemental data

Supplemental data

Supplemental data

## Abbreviations Used

ANCOVA: analysis of covariance
CI: confidence interval
GDM: gestational diabetes mellitus
NICU: neonatal intensive care unit
PRC: partial regression coefficient
*p* value: significant probability

## References

[1] Cohen LS, Altshuler LL, Harlow BL, et al. Relapse of major depression during pregnancy in women who maintain or discontinue antidepressant treatment. JAMA 2006;295(5):499–507; doi: 10.1001/jama.295.5.499 16449615

[2] Trost SL, Beauregard JL, Smoots AN, et al. Preventing pregnancy-related mental health deaths: Insights from 14 US maternal mortality review committees, 2008–17. Health Aff (Millwood) 2021;40(10):1551–1559; doi: 10.1377/hlthaff.2021.00615 34606354 PMC11135281

[3] Huybrechts KF, Palmsten K, Avorn J, et al. Antidepressant use in pregnancy and the risk of cardiac defects. N Engl J Med 2014;370(25):2397–2407; doi: 10.1056/NEJMoa1312828 24941178 PMC4062924

[4] Yakuwa N, Takahashi K, Anzai T, et al. Pregnancy outcomes with exposure to second-generation antipsychotics during the first trimester. J Clin Psychiatry 2022;83(4); doi: 10.4088/JCP.21m14081 35687862

[5] Battle CL, Salisbury AL, Schofield CA, et al. Perinatal antidepressant use: Understanding women's preferences and concerns. J Psychiatr Pract 2013;19(6):443–453; doi: 10.1097/01.pra.0000438183.74359.46 24241498 PMC4277178

[6] McElhatton PR. The effects of benzodiazepine use during pregnancy and lactation. Reprod Toxicol 1994;8(6):461–475; doi: 10.1016/0890-6238(94)90029-9 7881198

[7] Glover V. Prenatal stress and its effects on the fetus and the child: Possible underlying biological mechanisms. Adv Neurobiol 2015;10:269–283; doi: 10.1007/978-1-4939-1372-5_13 25287545

[8] McFarland J, Salisbury AL, Battle CL, et al. Major depressive disorder during pregnancy and emotional attachment to the fetus. Arch Womens Ment Health 2011;14(5):425–434; doi: 10.1007/s00737-011-0237-z 21938509 PMC3248759

[9] Viguera AC, Whitfield T, Baldessarini RJ, et al. Risk of recurrence in women with bipolar disorder during pregnancy: Prospective study of mood stabilizer discontinuation. Am J Psychiatry 2007;164(12):1817–1824; quiz 923; doi: 10.1176/appi.ajp.2007.06101639 18056236

[10] Patorno E, Huybrechts KF, Bateman BT, et al. Lithium use in pregnancy and the risk of cardiac malformations. N Engl J Med 2017;376(23):2245–2254; doi: 10.1056/NEJMoa1612222 28591541 PMC5667676

[11] Vajda FJ, O'Brien TJ, Hitchcock A, et al. Critical relationship between sodium valproate dose and human teratogenicity: Results of the Australian register of anti-epileptic drugs in pregnancy. J Clin Neurosci 2004;11(8):854–858; doi: 10.1016/j.jocn.2004.05.003 15519862

[12] Meador KJ, Baker GA, Browning N, et al. Cognitive function at 3years of age after fetal exposure to antiepileptic drugs. N Engl J Med 2009;360(16):1597–1605; doi: 10.1056/NEJMoa0803531 19369666 PMC2737185

[13] Park Y, Hernandez-Diaz S, Bateman BT, et al. Continuation of atypical antipsychotic medication during early pregnancy and the risk of gestational diabetes. Am J Psychiatry 2018;175(6):564–574; doi: 10.1176/appi.ajp.2018.17040393 29730938 PMC5988929

[14] Salvi V, Grua I, Cerveri G, et al. The risk of new-onset diabetes in antidepressant users—A systematic review and meta-analysis. PLoS One 2017;12(7):e0182088; doi: 10.1371/journal.pone.0182088 28759599 PMC5536271

[15] Hutchison SM, Masse LC, Pawluski JL, et al. Perinatal selective serotonin reuptake inhibitor (SSRI) effects on body weight at birth and beyond: A review of animal and human studies. Reprod Toxicol 2018;77:109–121; doi: 10.1016/j.reprotox.2018.02.004 29447847

[16] Isaac R, Boura-Halfon S, Gurevitch D, et al. Selective serotonin reuptake inhibitors (SSRIs) inhibit insulin secretion and action in pancreatic beta cells. J Biol Chem 2013;288(8):5682–5693; doi: 10.1074/jbc.M112.408641 23275337 PMC3581374

[17] Lupattelli A, Barone-Adesi F, Nordeng H. Association between antidepressant use in pregnancy and gestational diabetes mellitus: Results from the Norwegian Mother, Father and Child Cohort Study. Pharmacoepidemiol Drug Saf 2022;31(2):247–256; doi: 10.1002/pds.5388 34817916

[18] Wang XY, Ying XH, Jiang HY. Antidepressant use during pregnancy and the risk for gestational diabetes: A systematic review and meta-analysis. J Matern Fetal Neonatal Med 2023;36(1):2162817; doi: 10.1080/14767058.2022.2162817 36599445

[19] Ruohomaki A, Toffol E, Upadhyaya S, et al. The association between gestational diabetes mellitus and postpartum depressive symptomatology: A prospective cohort study. J Affect Disord 2018;241:263–268; doi: 10.1016/j.jad.2018.08.070 30138811

[20] Christensen MH, Andersen MS, Rubin KH, et al. Psychiatric morbidity in women with previous gestational diabetes mellitus: A nationwide register-based cohort study. Diabetes Care 2023;46(5):1076–1084; doi: 10.2337/dc22-1961 36928320

[21] Tasnim S, Auny FM, Hassan Y, et al. Antenatal depression among women with gestational diabetes mellitus: A pilot study. Reprod Health 2022;19(1):71; doi: 10.1186/s12978-022-01374-1 35305655 PMC8934461

[22] Azami M, Badfar G, Soleymani A, et al. The association between gestational diabetes and postpartum depression: A systematic review and meta-analysis. Diabetes Res Clin Pract 2019;149:147–155; doi: 10.1016/j.diabres.2019.01.034 30735772

[23] Kim SY, England L, Wilson HG, et al. Percentage of gestational diabetes mellitus attributable to overweight and obesity. Am J Public Health 2010;100(6):1047–1052; doi: 10.2105/AJPH.2009.172890 20395581 PMC2866592

[24] Harrington RA, Lee LC, Crum RM, et al. Prenatal SSRI use and offspring with autism spectrum disorder or developmental delay. Pediatrics 2014;133(5):e1241–e1248; doi: 10.1542/peds.2013-3406 24733881 PMC4006441

[25] Kim JY, Son MJ, Son CY, et al. Environmental risk factors and biomarkers for autism spectrum disorder: An umbrella review of the evidence. Lancet Psychiatry 2019;6(7):590–600; doi: 10.1016/S2215-0366(19)30181-6 31230684

[26] Leshem R, Bar-Oz B, Diav-Citrin O, et al. Selective serotonin reuptake inhibitors (SSRIs) and serotonin norepinephrine reuptake inhibitors (SNRIs) during pregnancy and the risk for autism spectrum disorder (ASD) and Attention deficit hyperactivity disorder (ADHD) in the Offspring: A true effect or a bias? A systematic review & meta-analysis. Curr Neuropharmacol 2021;19(6):896–906; doi: 10.2174/1570159X19666210303121059 33655866 PMC8686301

[27] Petersen I, Evans S, Nazareth I. Prenatal exposure to selective serotonin reuptake inhibitors and autistic symptoms in young children: Another red herring? Br J Psychiatry 2014;205(2):105–106; doi: 10.1192/bjp.bp.113.141721 25252319

